# Impact of deviation from guideline recommended treatment on breast cancer survival in Asia

**DOI:** 10.1038/s41598-020-58007-5

**Published:** 2020-01-28

**Authors:** Peh Joo Ho, Samuel Guan Wei Ow, Yirong Sim, Jenny Liu, Swee Ho Lim, Ern Yu Tan, Su-Ming Tan, Soo Chin Lee, Veronique Kiak-Mien Tan, Yoon-Sim Yap, Wen Yee Chay, Benita Kiat Tee Tan, Fuh Yong Wong, Jingmei Li, Mikael Hartman

**Affiliations:** 1 Human Genetics, Genome Institute of Singapore, Singapore, 138672 Singapore; 2 Saw Swee Hock School of Public Health, National University of Singapore and National University Health System, Singapore, 117549 Singapore; 3 Department of Haematology – Oncology, National University Cancer Institute Singapore (NCIS), Singapore, 119074 Singapore; 4 Division of Surgical Oncology, National Cancer Centre Singapore, Singapore, Singapore; 5 Department of General Surgery, Singapore General Hospital, Singapore, Singapore; 6 KK Women’s and Children’s Hospital, KK Breast Department, Singapore, 229899 Singapore; 7 Department of General Surgery, Tan Tock Seng Hospital, Singapore, 308433 Singapore; 8 Division of Breast Surgery, Department of Surgery, Changi General Hospital, Singapore, Singapore; 9 Division of Medical Oncology, National Cancer Centre Singapore, Singapore, Singapore; 10 Karolinska Institutet, Department of Medical Epidemiology and Biostatistics, Box 281, 171 77 Stockholm, Sweden; 11 Department of Surgery, Yong Loo Lin School of Medicine, National University of Singapore and National University Health System, Singapore, 119228 Singapore

**Keywords:** Epidemiology, Risk factors

## Abstract

Breast cancer survival has improved with significant progress in treatment and disease management. However, compliance with treatment varies. Treatment guidelines for older patients are unclear. We aim to identify predictors of noncompliance with recommended therapy in a large breast cancer population and assess the impact of noncompliance on survival. Our study included 19,241 non-metastatic female breast cancer patients, of whom 3,158 (16%) died within 10 years post-diagnosis (median survival = 5.8 years). We studied the association between treatment noncompliance and factors with logistic regression, and the impact of treatment noncompliance on survival with a flexible parametric survival model framework. The highest proportion of noncompliance was observed for chemotherapy (18%). Predictors of noncompliance with chemotherapy, radiotherapy and endocrine therapy included age, tumor size, nodal involvement and subtype (except radiotherapy). Factors associated with not receiving surgery included age and subtype. Treatment noncompliance was associated with worse overall survival for surgery (HR: 2.26 [1.80–2.83]), chemotherapy (1.25 [1.11–1.41]), radiotherapy (2.28 [1.94–2.69]) and endocrine therapy (1.70 [1.41–2.04]). Worse survival was similarly observed in older patients for whom guidelines generally do not apply. Our results highlight the importance of following appropriate treatment as recommended by current guidelines. Older patients may benefit from similar recommendations.

## Introduction

Breast cancer is the leading type of cancer among Asian women, with an increasing number of cases diagnosed every year^1^. Between 2003 and 2008, there were more than 2 million women living with breast cancer in South-East Asia, where a population of over 650 million women resides^2^. As breast cancer is common, the number of lives claimed by the disease is high. On average ~100,000 deaths from breast cancer were recorded every year in the same region^3^. With significant progress in treatment and disease management, a growing number of women are surviving breast cancer^4^. However, breast cancer survival can vary between countries. While 90% of patients in the United States live at least five years after the cancer is found^5^, the corresponding proportion is lower in South-East Asia. In a report by Bhoo-Pathy *et al*., five-year overall survival rates were estimated to range from 58.5% to 75.8% in South-East Asia^6^.

Several professional organizations and consensus groups exist to translate evidence-based medicine into recommendations for best patient care^7^. Examples of providers of such clinical practice guidelines include the American Society of Clinical Oncology (ASCO), the European Society for Medical Oncology (ESMO), the U.S. National Comprehensive Cancer Network (NCCN) and the St. Gallen International Breast Cancer Consensus Conference^7–9^. Guideline-based treatment has been shown to improve overall survival in breast cancer patients^10–13^. For example, breast cancer patients who underwent recommended radiotherapy were up to four times less likely to die from the disease than patients who did not follow the recommended treatment^12–14^. Similarly, breast cancer patients who complied to recommended endocrine therapy were two times less likely to die from any cause, compared to patients who were noncompliant with recommended treatment^12,15^.

Despite the established survival benefit, some patients decline evidence-based treatment recommendations offered by their physicians^16^. Attitudes towards health is known to vary between cultures, which may have a strong impact on the uptake of recommended treatment and survival outcome^17,18^. Horne *et al*. defined compliance as “the extent to which the patient’s behavior matches the prescriber’s recommendations”^19^. In this study, we aim to identify factors associated with compliance with recommended treatment in an Asian breast cancer population and assess the impact of treatment compliance on survival. As treatment guidelines are unclear for older breast cancer patients^20^, we also examined whether recommended treatment confer a survival benefit to patients over 70 years of age.

## Methods

### Study population

Hospital-based registry data on 20,999 citizens and permanent residents of Singapore diagnosed with breast cancer (ICD 9: 174*; ICD 10: C50*) between 2005 and 2015 was obtained from six major restructured public hospitals in Singapore (Changi General Hospital [CGH], KK Women’s and Children’s Hospital [KKH], National Cancer Center Singapore [NCCS], National University Hospital [NUH], Singapore General Hospital [SGH], and Tan Tock Seng Hospital [TTSH]). Patients who were diagnosed with metastatic breast cancer (*n* = 1,644), who were male (*n* = 26), whose date of birth was not reported (*n* = 6), or who had missing information on death date (*n* = 82) were excluded (Fig. S1). Ethical approval for using the de-identified pooled data was obtained from the National Healthcare Group Domain Specific Review Board (NHG DSRB Ref: 2013/01085 and CIRB Ref: 2016/3010). The institutional review boards of the respective hospitals involved in this study had approved the conduct of this study without the need for informed consent.

### Compliance with treatment guidelines

The restructured public hospitals in Singapore generally follow the guidelines of the NCCN and the St Gallen 2005 consensus (Table 1)^9,20^. For each treatment (chemotherapy, radiotherapy and endocrine therapy), patients were classified into three groups: (1) started recommended treatment [compliant], (2) did not start recommended treatment [noncompliant], or (3) treatment not recommended [compliant]. All patients were non-metastatic at diagnosis and should be recommended surgery (i.e. started recommended treatment [compliant] or did not start recommended treatment [noncompliant]).

**Table 1:** Recommended treatment for women diagnosed with non-metastatic breast cancer as adapted from the St Gallen 2005 consensus.

Treatment	Criteria
Surgery	Non-metastatic breast cancer patients
Chemotherapy	If a patient meets the following criteria the patient would be considered indicated for chemotherapy: ● Positive nodes ● Tumor size from pathology report > 20 mm or attached to chest wall ● Tumor size > 5 mm with one or more of the following high risk criteria ○ Grade 3 tumor ○ Estrogen receptor status negative ○ HER2-positive ○ Age ≤ 35 years
Radiotherapy	Patients who fulfill either or both criteria: ● Positive nodes ● Breast conserving surgery ● Tumor size > 50 mm or attached to chest wall
Endocrine therapy	Patients who are estrogen or progesterone receptor status positive

### Demographic and clinical characteristics

Demographic and clinical characteristics were extracted from hospital-based registries, which include age at diagnosis (<50, 50–69, ≥70 years), ethnicity (Chinese, Malay, Indian, other), year of diagnosis (2005–2010, 2011–2015), pre-surgical tumor size according to TNM classification (≤20 mm, 21–50 mm, >50 mm, attached to chest wall, unknown), pathological TNM nodal stage (N0, N1, N2, N3, unknown), pathological tumor stage (*in situ*, stage I, II, III, unknown), grade (well-differentiated, moderately-differentiated, poorly-differentiated, unknown), estrogen receptor status (ER: positive, negative, unknown), progesterone receptor status (PR: positive, negative, unknown), human epidermal growth factor receptor 2 status (HER2: positive, negative, unknown), proxy tumor subtypes (luminal A [ER-positive/PR-positive, and well- or moderately-differentiated], luminal B (HER2-positive) [ER-positive/PR-positive, HER2-positive, and poorly-differentiated], luminal B (HER2-negative) [ER-positive/PR-positive, HER2-negative, and poorly-differentiated], HER2-enriched [ER-negative, PR-negative, and HER2-positive], basal [ER-negative, PR-negative, and HER2-negative], unknown)^9^.

### Outcome of interest

The primary outcome of interest was overall survival, with time since diagnosis in years as the underlying time scale. Patients were followed up until death, or censored due to either loss of follow-up or end of study period (ten years after date of diagnosis). Information on death was verified by each hospital with the National Registry of Births and Deaths in Singapore through National Registry Disease Office^21^. Date of last follow-up for breast cancer patients verified as alive for SGH and NCCS was the date of last visit to the clinic. Date of last follow-up for other institutions was defined as the date on which the patient’s vital status was verified with National Registry of Births and Deaths (KKH: 30-June-2017, NUH: 30-April-2017, TTSH: 30-April-2018, CGH: 16-April-2018).

### Statistical analysis

Bivariate associations between vital status (dead or alive) and patient-related characteristics (demographic, clinical and treatment) were evaluated using the Chi-square test. To identify patient-related characteristics associated with the compliance with recommended treatment, logistic regression was used to estimate the odds ratios (OR) and corresponding 95% confidence intervals.

To examine the effect of treatment compliance on survival, mortality rate was calculated. This risk estimate was defined as the number of deaths divided by risk time (total person-years [PY] for which the patients were alive), modeled using flexible parametric survival model, with cubic splines function for baseline mortality. The associations between individual treatments and mortality rates (unadjusted) was first modelled assuming proportion hazards throughout the follow-up time. A flexible parametric survival model framework was then used to model time-dependent effects (STATA version 8, command: stpm2), hazard ratios (HR) and corresponding 95% confidence intervals were estimated. The Akaike Information Criterion (AIC) statistic was used to optimize the number of internal knots (i.e. degrees of freedom of the baseline model) and the number of knots for the time varying component knots (i.e. degrees of freedom of the time varying component). Degrees of freedom used in the respective flexible parametric survival models are shown in Supplementary Table 1. Kaplan-Meier survival curves were plotted to graphically represent survival over time (R Version 3.4.3, package: survival).

## Results

A total of 19,241 female breast cancer patients with 3,158 (16%) deaths occurring within 10 years of diagnosis were included in this study (Table 2). The median survival time was 5.8 years (interquartile range: 3.3–8.7). The majority were Chinese (*n* = 15,419, 80%), 2,108 (11%) were Malay, and 1,113 (6%) were Indian. Thirty-five percent (*n* = 6,699) of our patients were diagnosed before age 50, 52% (*n* = 10,027) between 50 and 69, and 13% (*n* = 2,515) at age 70 and above. Fifteen percent (*n* = 2,884), 27% (*n* = 5,183), 33% (*n* = 6,312), and 15% (*n* = 2,861) of our patients were diagnosed with *in situ*, stage I to III breast cancer, respectively. The largest proportion of our patients were of luminal A proxy subtype (*n* = 5,934, 31%), followed by luminal B [HER2-negative] (*n* = 2,610, 14%), luminal B [HER2-positive] (*n* = 2,189, 11%), HER2-enriched (*n* = 1,491, 8%), and basal (*n* = 1,658, 9%). The highest proportion of noncompliance with treatment was observed for chemotherapy 18% (*n* = 3,482), followed by radiotherapy (8%, *n* = 1,614), surgery (8%, *n* = 1604), and endocrine therapy (8%, *n* = 1,533) (Table 2).

**Table 2:** Demographic, tumor characteristic and treatment variables of 19,241 women diagnosed with breast cancer between 2005 and 2015 by vital status.

	Vital status		P-value
	Alive	Dead	
	n = 16,083 (84%)	n = 3,158 (16%)	
Median survival time (IQR)	6.3 (3.8–9.1)	3.2 (1.7–5.4)	<0.001
**Demographics**			
Ethnicity			
Chinese	13,101 (85%)	2,318 (15%)	<0.001
Malay	1,588 (75%)	520 (25%)	
Indian	897 (81%)	216 (19%)	
Other	497 (83%)	104 (17%)	
**Median age (IQR)**			
Age group, years	53 (46–62)	59 (50–72)	<0.001
<50	5,941 (89%)	758 (11%)	<0.001
50–69	8,566 (85%)	1,461 (15%)	
≥70	1,576 (63%)	939 (37%)	
**Year of diagnosis**			
2005–2010	8,257 (80%)	2,115 (20%)	<0.001
2011–2015	7,826 (88%)	1,043 (12%)	
**Center**			
CGH	995 (79%)	258 (21%)	<0.001
KKH	3,659 (89%)	455 (11%)	
NUH	2,218 (84%)	425 (16%)	
SGH and NCCS	7,575 (82%)	1,609 (18%)	
TTSH	1,636 (80%)	411 (20%)	
**Tumor characteristics**			
**Pre-surgical tumor size**			
≤20 mm	7,518 (93%)	608 (7%)	<0.001
21–50 mm	4,823 (82%)	1,074 (18%)	
>50 mm	1,684 (79%)	444 (21%)	
Attached to chest wall	494 (57%)	379 (43%)	
Unknown	1,564 (71%)	653 (29%)	
**TNM nodal stage**			
N0	10,628 (92%)	928 (8%)	<0.001
N1	2,823 (83%)	564 (17%)	
N2	1,083 (74%)	383 (26%)	
N3	623 (60%)	422 (40%)	
Unknown	926 (52%)	861 (48%)	
**Tumor stage**			
*In situ*	2,734 (95%)	150 (5%)	<0.001
I	4,857 (94%)	326 (6%)	
II	5,464 (87%)	848 (13%)	
III	1,933 (68%)	928 (32%)	
Unknown	1,095 (55%)	906 (45%)	
**Grade**			
Well-differentiated	2,522 (92%)	218 (8%)	<0.001
Moderately-differentiated	5,925 (88%)	788 (12%)	
Poorly-differentiated	6,397 (80%)	1,552 (20%)	
Unknown	1,239 (67%)	600 (33%)	
Estrogen receptor status			
Positive	11,069 (86%)	1,786 (14%)	<0.001
Negative	3,275 (77%)	985 (23%)	
Unknown	1,739 (82%)	387 (18%)	
Progesterone receptor status			
Positive	9,577 (87%)	1,484 (13%)	<0.001
Negative	4,631 (79%)	1,255 (21%)	
Unknown	1,875 (82%)	419 (18%)	
**HER2 status**			
Positive	3,020 (82%)	675 (18%)	0.001
Negative	8,975 (84%)	1,743 (16%)	
Unknown	4,088 (85%)	740 (15%)	
**Proxy subtype**			
Luminal A	5,342 (90%)	592 (10%)	<0.001
Luminal B, HER2-negative	2,091 (80%)	519 (20%)	
Luminal B, HER2-positive	1,810 (83%)	379 (17%)	
HER2-enriched	1,198 (80%)	293 (20%)	
Basal	1,217 (73%)	441 (27%)	
Unknown	4,425 (83%)	934 (17%)	
**Treatment**			
**Surgery**			
Yes	15,295 (87%)	2,342 (13%)	<0.001
No	788 (49%)	816 (51%)	
**Chemotherapy**			
Yes	6,251 (83%)	1,301 (17%)	<0.001
No	2,521 (72%)	961 (28%)	
Not recommended	7,311 (89%)	896 (11%)	
**Radiotherapy**			
Yes	7,013 (87%)	1,044 (13%)	<0.001
No	1,180 (73%)	434 (27%)	
Not recommended	6,243 (91%)	635 (9%)	
Unknown	1,647 (61%)	1,045 (39%)	
**Endocrine therapy**			
Yes	9,217 (86%)	1,501 (14%)	<0.001
No	1,305 (85%)	228 (15%)	
Not recommended	2,843 (77%)	831 (23%)	
Unknown	2,718 (82%)	598 (18%)	

TNM nodal stage: post-surgery if surgery is done.

Tumor stage: post-surgery, patients without surgery are classified as unknown.

### Factors associated with treatment noncompliance in patients who were recommended treatment

Proxy subtype had the strongest association with not undergoing surgery (luminal B [HER2-positive], HER2-enriched, and basal vs. luminal A, OR: 4.08 [2.72–6.12], 2.50 [1.52–4.12] and 2.03 [1.24–3.35] respectively), followed by age at diagnosis (<50 and ≥70 vs. 50–69 years, OR: 1.57 [1.30–1.89] and 2.94 [2.34–3.68] respectively) and pre-surgical tumor size (21–50 mm and >50 mm vs. <20 mm, OR: 2.23 [1.85–2.68] and 2.54 [1.93–3.34] respectively) (Table 3).

**Table 3:** The odds of not receiving treatment with respect to demographic characteristics and treatment was studied using logistic model.

	Surgery*, *n* = 19,241		Chemotherapy*, *n* = 11,034		Radiotherapy*, *n* = 9,671		Endocrine therapy*, *n* = 12,251	
	Unadjusted	Adjusted^	Unadjusted	Adjusted^	Unadjusted	Adjusted^	Unadjusted	Adjusted^
	OR (95%CI)	OR (95%CI)	OR (95%CI)	OR (95%CI)	OR (95%CI)	OR (95%CI)	OR (95%CI)	OR (95%CI)
**Ethnicity**								
Chinese	1.00 (Reference)	1.00 (Reference)	1.00 (Reference)	1.00 (Reference)	1.00 (Reference)	1.00 (Reference)	1.00 (Reference)	1.00 (Reference)
Malay	1.12 (0.87 to 1.43)	1.25 (0.95 to 1.63)	0.70 (0.62 to 0.80)	0.90 (0.78 to 1.04)	1.23 (1.05 to 1.44)	1.10 (0.92 to 1.30)	0.98 (0.83 to 1.17)	1.07 (0.89 to 1.28)
Indian	1.27 (0.93 to 1.73)	1.33 (0.95 to 1.85)	1.08 (0.92 to 1.28)	1.25 (1.04 to 1.52)	1.40 (1.14 to 1.72)	1.26 (1.01 to 1.58)	0.98 (0.78 to 1.24)	1.02 (0.79 to 1.30)
Other	1.16 (0.75 to 1.79)	1.31 (0.82 to 2.09)	0.90 (0.72 to 1.14)	0.91 (0.70 to 1.18)	0.95 (0.69 to 1.30)	0.80 (0.57 to 1.13)	1.04 (0.76 to 1.40)	0.97 (0.70 to 1.34)
**Age at diagnosis**, **years**								
<50	1.54 (1.29 to 1.84)	1.57 (1.30 to 1.89)	0.87 (0.79 to 0.95)	0.77 (0.69 to 0.85)	0.83 (0.74 to 0.94)	0.80 (0.70 to 0.91)	1.25 (1.11 to 1.40)	1.17 (1.03 to 1.32)
50–69	1.00 (Reference)	1.00 (Reference)	1.00 (Reference)	1.00 (Reference)	1.00 (Reference)	1.00 (Reference)	1.00 (Reference)	1.00 (Reference)
≥70	2.77 (2.25 to 3.41)	2.94 (2.34 to 3.68)	9.92 (8.61 to 11.42)	13.96 (11.94 to 16.32)	3.17 (2.72 to 3.69)	3.27 (2.75 to 3.88)	1.15 (0.98 to 1.36)	1.08 (0.91 to 1.29)
**Year of diagnosis**								
2005–2010	1.00 (Reference)	1.00 (Reference)	1.00 (Reference)	1.00 (Reference)	1.00 (Reference)	1.00 (Reference)	1.00 (Reference)	1.00 (Reference)
2011–2015	0.75 (0.64 to 0.88)	1.15 (0.96 to 1.38)	1.20 (1.11 to 1.31)	1.15 (1.05 to 1.27)	1.11 (1.00 to 1.23)	1.05 (0.93 to 1.19)	1.05 (0.94 to 1.17)	1.14 (1.02 to 1.28)
**Pre-surgical tumor size**								
≤20 mm	1.00 (Reference)	1.00 (Reference)	1.00 (Reference)	1.00 (Reference)	1.00 (Reference)	1.00 (Reference)	1.00 (Reference)	1.00 (Reference)
21–50 mm	1.45 (1.23 to 1.72)	2.23 (1.85 to 2.68)	0.63 (0.57 to 0.69)	0.61 (0.54 to 0.68)	1.21 (1.07 to 1.36)	1.02 (0.88 to 1.18)	0.51 (0.45 to 0.58)	0.67 (0.58 to 0.77)
>50 mm	1.24 (0.97 to 1.58)	2.54 (1.93 to 3.34)	0.58 (0.51 to 0.67)	0.59 (0.50 to 0.70)	1.25 (1.06 to 1.47)	0.92 (0.77 to 1.12)	0.43 (0.35 to 0.54)	0.46 (0.37 to 0.58)
Attached to chest wall			0.54 (0.44 to 0.65)	0.31 (0.24 to 0.39)	1.28 (1.00 to 1.64)	0.81 (0.61 to 1.07)	0.52 (0.39 to 0.70)	0.55 (0.39 to 0.76)
**TNM nodal stage**								
N0	—	—	1.00 (Reference)	1.00 (Reference)	1.00 (Reference)	1.00 (Reference)	1.00 (Reference)	1.00 (Reference)
N1			0.51 (0.46 to 0.57)	0.35 (0.31 to 0.40)	2.55 (2.26 to 2.89)	2.53 (2.20 to 2.92)	0.24 (0.19 to 0.29)	0.30 (0.25 to 0.38)
N2			0.36 (0.31 to 0.42)	0.26 (0.21 to 0.30)	0.79 (0.64 to 0.96)	0.71 (0.57 to 0.89)	0.25 (0.19 to 0.34)	0.36 (0.27 to 0.49)
N3			0.51 (0.43 to 0.60)	0.32 (0.26 to 0.39)	1.46 (1.21 to 1.78)	1.22 (0.98 to 1.51)	0.57 (0.43 to 0.74)	0.67 (0.50 to 0.90)
**Proxy subtype**								
Luminal A	1.00 (Reference)	1.00 (Reference)	1.00 (Reference)	1.00 (Reference)	1.00 (Reference)	1.00 (Reference)	1.00 (Reference)	1.00 (Reference)
Luminal B, HER2-negative	1.28 (0.77 to 2.13)	1.22 (0.73 to 2.05)	1.15 (1.01 to 1.31)	0.72 (0.62 to 0.85)	1.10 (0.93 to 1.30)	1.08 (0.90 to 1.30)	1.20 (1.01 to 1.42)	1.49 (1.25 to 1.78)
Luminal B, HER2-positive	4.17 (2.80 to 6.21)	4.08 (2.72 to 6.12)	1.05 (0.92 to 1.20)	0.57 (0.48 to 0.67)	1.16 (0.97 to 1.39)	1.16 (0.95 to 1.42)	1.45 (1.22 to 1.73)	1.80 (1.50 to 2.16)
HER2-enriched	2.71 (1.66 to 4.44)	2.50 (1.52 to 4.12)	0.88 (0.75 to 1.02)	0.41 (0.34 to 0.50)	1.08 (0.87 to 1.34)	1.04 (0.82 to 1.32)	—	—
Basal	2.37 (1.45 to 3.89)	2.03 (1.24 to 3.35)	0.93 (0.80 to 1.08)	0.42 (0.35 to 0.50)	0.98 (0.80 to 1.20)	0.94 (0.75 to 1.17)	—	—

*Women who started treatment was used as the reference category of the outcome, hence an odds ratio (OR) value above 1 implies that the exposure category (e.g. Indian) has higher odds of not starting recommended treatment than the reference level of the exposure factor (e.g. Chinese). CI: Confidence interval. ^Additionally adjusted for site.

Factors associated with chemotherapy noncompliance were ethnicity (Indian vs. Chinese, OR: 1.25 [1.04–1.52]), older age at diagnosis (≥70 vs. 50–59 years, OR: 13.96 [11.94–16.32]), and year of diagnosis (2011–2015 vs. 2005–2010, OR: 1.15 [1.05–1.27]) (Table 3). Pre-surgical tumor size (21–50 mm, >50 mm, or attached to chest wall vs. <20 mm), nodal stage (N1, N2, or N3 vs. N0), and proxy subtype (luminal B [HER2-negative], luminal B [HER2-positive], HER2-enriched or basal vs. luminal A), were associated with compliance with recommended chemotherapy (OR <1.00 and P < 0.001 for all comparison).

Radiotherapy noncompliance was associated with ethnicity (Indian vs. Chinese, OR: 1.26 [1.01–1.58]), age at diagnosis (≥70 vs. 50–59 years, OR: 3.27 [2.75–3.88]), and nodal stage (N1 vs N0, OR: 2.53 [2.20–2.92]) (Table 3). Factors associated with radiotherapy compliance were pre-surgical tumor size (21–50 mm, >50 mm, or attached to chest wall vs. <20 mm: OR <1.00 and P < 0.001 for all comparison) and nodal stage (N2 vs N0, OR: 0.71 [0.57–0.89]).

Younger age at diagnosis (<50 vs. 50–59 years, OR: 1.17 [1.03–1.32]), year of diagnosis (2011–2015 vs. 2005–2010, OR: 1.14 [1.02–1.28]), and proxy subtype (luminal B [HER2-negative] and luminal B [HER2-positive] vs. luminal A, OR: 1.49 [1.25–1.78] and 1.80 [1.50–2.16], respectively) were associated with endocrine therapy noncompliance (Table 3). Pre-surgical tumor size (21–50 mm, >50 mm, or attached to chest wall vs. <20 mm) and nodal stage (N1, N2, or N3 vs. N0) were associated with compliance with recommended endocrine therapy (OR <1.00 and P < 0.001 for all comparisons).

### Mortality rates by treatment compliance

**Figure 1:**
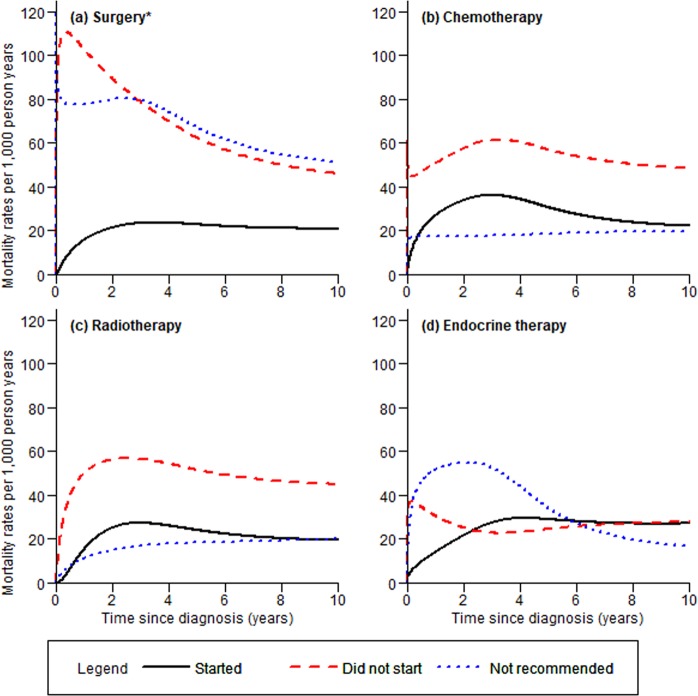
Unadjusted mortality rates of breast cancer patients by treatments; (**a**) surgery, (**b**) chemotherapy, (**c**) radiotherapy, and (**d**) endocrine therapy. *Patients who had tumors attached to chest wall or whose pre-surgical tumor size were not known were represented by the blue dotted line.

Not undergoing recommended surgery resulted in worse survival, particularly in the first year after breast cancer diagnosis (Fig. 1a). A larger proportion of patients who did not start recommended chemotherapy or radiotherapy died compared to patients who started recommended treatment and patients for whom treatment was not recommended (Fig. 1b,c). While mortality rates for patients who were not recommended treatment (vs. started treatment) were slightly lower for chemotherapy and radiotherapy, the mortality rate for patients who were not recommended endocrine therapy (i.e. patients who are ER- and PR-negative) was higher than the corresponding rate for patients who started endocrine therapy within ~6 years post-diagnosis (Fig. 1d).

### Overall survival in patients aged <70 years

Noncompliance with recommended therapy was associated with worse overall survival for surgery (HR: 2.2 [1.80–2.83]), chemotherapy (HR: 1.25 [1.11–1.41]), radiotherapy (HR: 2.28 [1.94–2.67]) and endocrine therapy (HR: 1.70 [1.41–2.04]) (Table 4). Largest differences (HR ≥ 9.66, P < 0.001) in overall survival was observed in patients who did not undergo surgery across all proxy subtypes. However, the effect was diminished for luminal B [HER2-negative] (HR: 6.43 [2.62–15.80]), basal (HR: 5.46 [2.05–14.54]) and luminal A (HR: 2.85 [1.31–6.18]), and was not significant for luminal B [HER2-positive] and HER2-enriched, upon adjusting for other treatments, tumor characteristics and patient’s demographics.

**Table 4:** The associations between demographic, clinical, and treatment variables and overall survival using the flexible parametric survival model, stratified by proxy subtype, in patients aged <70 years.

	All patients aged < 70 years		Luminal A		Luminal B, HER2-negative	
	Unadjusted	Adjusted^	Unadjusted	Adjusted^	Unadjusted	Adjusted^
	HR (95%CI)	HR (95%CI)	HR (95%CI)	HR (95%CI)	HR (95%CI)	HR (95%CI)
**Surgery**						
Yes	1.00 (Reference)	1.00 (Reference)	1.00 (Reference)	1.00 (Reference)	1.00 (Reference)	1.00 (Reference)
No	6.97 (6.28–7.74)	2.26 (1.80–2.83)	12.92 (9.30–17.95)	2.85 (1.31–6.18)	14.04 (9.78–20.14)	6.43 (2.62–15.80)
**Chemotherapy**						
Yes	1.00 (Reference)	1.00 (Reference)	1.00 (Reference)	1.00 (Reference)	1.00 (Reference)	1.00 (Reference)
No	1.51 (1.35–1.70)	1.25 (1.11–1.41)	1.51 (1.04–2.19)	1.57 (1.03–2.39)	1.19 (0.96–1.49)	1.12 (0.87–1.45)
Not recommended	0.47 (0.42–0.52)	0.69 (0.60–0.80)	0.64 (0.50–0.82)	0.60 (0.32–1.12)	0.14 (0.04–0.58)	0.35 (0.08–1.43)
**Radiotherapy**						
Yes	1.00 (Reference)	1.00 (Reference)	1.00 (Reference)	1.00 (Reference)	1.00 (Reference)	1.00 (Reference)
No	2.50 (2.13–2.94)	2.28 (1.94–2.67)	2.12 (1.45–3.12)	2.36 (1.53–3.64)	2.25 (1.70–2.98)	2.32 (1.68–3.21)
Not recommended	0.68 (0.58–0.79)	1.37 (1.14–1.64)	1.18 (0.89–1.56)	2.40 (1.60–3.61)	0.52 (0.39–0.70)	1.11 (0.73–1.67)
**Endocrine therapy**						
Yes	1.00 (Reference)	1.00 (Reference)	1.00 (Reference)	1.00 (Reference)	1.00 (Reference)	1.00 (Reference)
No	1.41 (1.17–1.69)	1.70 (1.41–2.04)	1.86 (1.29–2.69)	1.71 (1.15–2.53)	2.12 (1.55–2.90)	1.81 (1.28–2.55)
Not recommended	2.19 (1.95–2.45)	1.82 (1.63–2.04)	—	—	—	—
**Pre-surgical tumor size**						
≤20 mm	1.00 (Reference)	1.00 (Reference)	1.00 (Reference)	1.00 (Reference)	1.00 (Reference)	1.00 (Reference)
21–50 mm	2.89 (2.48–3.35)	1.78 (1.53–2.06)	1.92 (1.49–2.48)	1.40 (1.07–1.84)	2.84 (1.94–4.16)	1.83 (1.24–2.71)
>50 mm	4.05 (3.41–4.81)	2.66 (2.25–3.15)	2.95 (2.17–4.02)	2.30 (1.63–3.25)	4.01 (2.62–6.14)	2.24 (1.44–3.48)
Attached to chest wall	11.71 (9.83–13.93)	4.37 (3.63–5.21)	7.58 (5.38–10.70)	3.49 (2.37–5.13)	9.66 (6.14–15.20)	4.17 (2.59–6.71)
**TNM nodal stage**						
N0	1.00 (Reference)	1.00 (Reference)	1.00 (Reference)	1.00 (Reference)	1.00 (Reference)	1.00 (Reference)
N1	2.35 (2.00–2.74)	1.71 (1.43–2.05)	1.31 (0.94–1.83)	0.87 (0.43–1.76)	2.13 (1.51–2.99)	1.66 (1.07–2.59)
N2	3.75 (3.14–4.49)	2.98 (2.47–3.60)	2.89 (2.02–4.13)	2.03 (0.99–4.15)	3.09 (2.12–4.53)	2.63 (1.66–4.18)
N3	7.73 (6.60–9.04)	4.54 (3.76–5.49)	6.27 (4.39–8.95)	4.39 (2.13–9.06)	6.11 (4.38–8.54)	5.42 (3.52–8.34)
**Ethnicity**						
Chinese	1.00 (Reference)	1.00 (Reference)	1.00 (Reference)	1.00 (Reference)	1.00 (Reference)	1.00 (Reference)
Malay	2.08 (1.87–2.31)	1.56 (1.40–1.74)	2.65 (2.07–3.39)	1.78 (1.37–2.31)	1.88 (1.49–2.38)	1.59 (1.25–2.03)
Indian	1.47 (1.24–1.73)	1.15 (0.97–1.36)	1.39 (0.90–2.12)	1.28 (0.83–1.97)	1.18 (0.79–1.78)	0.87 (0.58–1.32)
Other	1.07 (0.82–1.38)	1.09 (0.84–1.41)	1.64 (0.99–2.72)	1.75 (1.05–2.93)	0.92 (0.45–1.85)	1.01 (0.50–2.06)
**Age at diagnosis**, **years**						
<50	0.73 (0.67–0.80)	0.78 (0.72–0.86)	0.44 (0.33–0.58)	0.42 (0.31–0.57)	0.80 (0.66–0.98)	0.85 (0.69–1.04)
50–69	1.00 (Reference)	1.00 (Reference)	1.00 (Reference)	1.00 (Reference)	1.00 (Reference)	1.00 (Reference)
**Year of diagnosis**						
2005–2010	1.00 (Reference)	1.00 (Reference)	1.00 (Reference)	1.00 (Reference)	1.00 (Reference)	1.00 (Reference)
2011–2015	0.96 (0.87–1.06)	0.98 (0.90–1.08)	0.89 (0.71–1.13)	0.96 (0.75–1.24)	0.81 (0.65–1.00)	0.92 (0.73–1.16)
**Surgery**						
Yes	1.00 (Reference)	1.00 (Reference)	1.00 (Reference)	1.00 (Reference)	1.00 (Reference)	1.00 (Reference)
No	10.27 (7.79–13.53)	2.55 (0.84–7.77)	11.59 (8.25–16.26)	1.62 (0.68–3.81)	9.66 (6.89–13.55)	5.46 (2.05–14.54)
**Chemotherapy**						
Yes	1.00 (Reference)	1.00 (Reference)	1.00 (Reference)	1.00 (Reference)	1.00 (Reference)	1.00 (Reference)
No	1.52 (1.16–2.01)	1.23 (0.90–1.67)	1.56 (1.10–2.20)	1.23 (0.83–1.81)	1.60 (1.22–2.10)	1.48 (1.08–2.03)
Not recommended	0.22 (0.07–0.76)	0.54 (0.16–1.85)	0.48 (0.23–0.99)	1.34 (0.60–3.00)	0.25 (0.05–1.15)	0.71 (0.15–3.47)
**Radiotherapy**						
Yes	1.00 (Reference)	1.00 (Reference)	1.00 (Reference)	1.00 (Reference)	1.00 (Reference)	1.00 (Reference)
No	2.17 (1.51–3.13)	3.11 (2.05–4.71)	2.43 (1.52–3.86)	2.64 (1.57–4.44)	2.15 (1.52–3.05)	2.68 (1.80–3.98)
Not recommended	0.58 (0.41–0.81)	2.79 (1.35–5.76)	0.56 (0.37–0.85)	1.91 (0.86–4.29)	0.42 (0.30–0.60)	0.64 (0.37–1.11)
**Endocrine therapy**						
Yes	1.00 (Reference)	1.00 (Reference)	—	—	—	—
No	1.46 (0.96–2.21)	1.79 (1.13–2.83)				
**Pre-surgical tumor size**						
≤20 mm	1.00 (Reference)	1.00 (Reference)	1.00 (Reference)	1.00 (Reference)	1.00 (Reference)	1.00 (Reference)
21–50 mm	3.39 (2.31–4.96)	2.06 (1.39–3.08)	3.46 (1.96–6.09)	1.95 (1.09–3.48)	2.29 (1.59–3.31)	1.72 (1.23–2.41)
>50 mm	3.64 (2.33–5.68)	2.22 (1.39–3.55)	4.52 (2.46–8.29)	2.61 (1.39–4.92)	4.48 (3.01–6.66)	3.16 (2.15–4.64)
Attached to chest wall	10.77 (6.96–16.67)	4.13 (2.54–6.70)	14.65 (7.92–27.11)	6.50 (3.39–12.48)	6.02 (3.81–9.53)	3.05 (1.90–4.88)
**TNM nodal stage**						
N0	1.00 (Reference)	1.00 (Reference)	1.00 (Reference)	1.00 (Reference)	1.00 (Reference)	1.00 (Reference)
N1	2.36 (1.64–3.38)	3.55 (1.73–7.28)	2.83 (1.79–4.46)	3.31 (1.49–7.34)	2.90 (1.95–4.30)	1.61 (1.10–2.35)
N2	4.35 (3.02–6.28)	7.21 (3.53–14.74)	4.13 (2.60–6.56)	5.89 (2.65–13.10)	4.78 (3.09–7.40)	2.63 (1.72–4.03)
N3	7.48 (5.01–11.18)	11.96 (5.71–25.05)	5.64 (3.58–8.88)	6.35 (2.84–14.21)	9.63 (6.50–14.29)	6.93 (4.69–10.25)
**Ethnicity**						
Chinese	1.00 (Reference)	1.00 (Reference)	1.00 (Reference)	1.00 (Reference)	1.00 (Reference)	1.00 (Reference)
Malay	1.76 (1.32–2.34)	1.16 (0.86–1.56)	1.82 (1.26–2.62)	1.74 (1.19–2.54)	1.80 (1.34–2.42)	1.44 (1.07–1.95)
Indian	1.78 (1.18–2.70)	1.11 (0.72–1.71)	1.67 (0.93–3.01)	1.32 (0.70–2.49)	1.05 (0.68–1.62)	0.76 (0.50–1.16)
Other	0.42 (0.13–1.31)	0.38 (0.12–1.19)	1.55 (0.72–3.30)	1.45 (0.67–3.17)	0.81 (0.35–1.84)	0.90 (0.40–2.04)
**Age at diagnosis**, **years**						
<50	0.49 (0.38–0.63)	0.54 (0.41–0.71)	0.78 (0.57–1.06)	0.93 (0.67–1.28)	1.10 (0.88–1.38)	1.17 (0.93–1.47)
50–69	1.00 (Reference)	1.00 (Reference)	1.00 (Reference)	1.00 (Reference)	1.00 (Reference)	1.00 (Reference)
**Year of diagnosis**						
2005–2010	1.00 (Reference)	1.00 (Reference)	1.00 (Reference)	1.00 (Reference)	1.00 (Reference)	1.00 (Reference)
2011–2015	0.85 (0.66–1.09)	0.91 (0.70–1.19)	0.92 (0.68–1.25)	1.01 (0.73–1.40)	1.08 (0.84–1.37)	1.20 (0.93–1.54)

HR: Hazards ratio, CI: Confidence interval. ^Adjusted for all variables listed in the table and site.

Chemotherapy noncompliance was associated with higher mortality within 10 years post-diagnosis, in patients with luminal A (HR: 1.57 [1.03–2.39]) and basal (HR: 1.48 [1.08–2.03]) proxy subtypes. Noncompliance with radiotherapy was associated with worse overall survival across all breast cancer proxy subtypes (adjusted P < 0.001). Patients who were noncompliant with recommended endocrine therapy had worse overall survival than compliant patients, in proxy subtypes luminal A (HR: 1.71 [1.15–2.53]), luminal B [HER2-negative] (HR: 1.81 [1.28–2.55]) and luminal B [HER2-positive] (HR: 1.79 [1.13–2.83]) tumors.

### Overall survival in older patients aged ≥70 years

**Figure 2:**
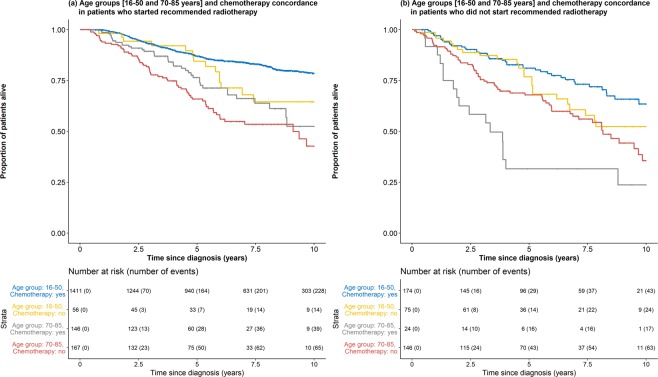
Overall survival of women diagnosed with and had surgery for breast cancer by age and compliance with chemotherapy by compliance with radiotherapy. The average life expectancy of females in Singapore is 85, thus patients aged 86 years and above were excluded from this analysis^42^. Survival curves for age group 51–69 and patients not recommend chemotherapy are not shown.

Patients aged ≥70 years who did not undergo surgery had a 1.74 [1.33–2.29] times increased risk of death within 10 years post-diagnosis as compared with those who had surgery (Supplementary Table 2). Noncompliance with recommended treatment was associated with worse overall survival (chemotherapy (HR: 1.31 [1.03–1.66]), radiotherapy (HR: 1.64 [1.30–2.07]) and endocrine therapy (HR: 1.57 [1.20–2.07])). In addition, the survival benefit associated with compliance to recommended chemotherapy was observed only when there is also compliance with radiotherapy (No vs Yes [reference], HR: 1.46 [0.98–2.17], log rank P = 0.061) (Fig. 2). Compliance with chemotherapy treatment was associated with worse survival in patients who were noncompliant with recommended radiotherapy (Yes vs No [reference], HR: 2.04 [1.19–3.49], log rank P = 0.008).

## Discussion

Treatment compliance, which has an impact on survival, was high for primary surgery, radiotherapy and endocrine therapy but low for chemotherapy. Factors associated with chemotherapy, radiotherapy and endocrine therapy noncompliance included older age, smaller pre-surgical tumor size, and lower nodal stage. Patients with luminal A proxy subtype were more likely to be noncompliant with chemotherapy than other proxy subtypes. Patients with luminal B [HER2-positive] or luminal B [HER2-negative] proxy subtype were more likely to be noncompliant with endocrine therapy than patients with luminal A proxy subtype. Noncompliance with recommended treatment (chemotherapy, radiotherapy, or endocrine therapy) was associated with a ~1.5 to 6-fold increased risk of dying within 10 years post-diagnosis. In patients aged ≥70 years who were recommended chemotherapy, noncompliance with recommended chemotherapy and radiotherapy were associated with worse survival.

### Factors associated with treatment noncompliance

In agreement with previous studies, tumor characteristics (pre-surgical tumor size, grade, proxy subtype) and age at diagnosis were associated with initiation of recommended treatment^22–24^. Many factors can potentially influence a patient’s decision to go along with recommended treatment: previous experiences and personal values^17^, fear of treatment-related side effects^18^, financial ability, age, education and health status, among others^19,20^. Although information on financial status was not available, the subjects in our study were patients at restructured hospitals and were eligible for subsidized treatment. It is thus less likely that any ethnic disparity observed was due to differences in financial resources^25^.

An ethnic disparity in recommended treatment uptake has also been observed by others; recommended treatment uptake is frequently lower in minority populations^26,27^. Although higher noncompliance with chemotherapy and radiotherapy were observed in Indian patients as compared with Chinese patients, the observed worse survival was not significant. To the contrary, we observed no disparate treatment uptake among Malay and Chinese ethnic groups where worse survival was observed in Malay patients (HR: 2.08 [1.87–2.31]). This worse survival of Malay patients remained (HR: 1.57 [1.41–1.75]) after accounting for compliance with treatment, pre-surgical tumor size, nodal status, age at diagnosis, year of diagnosis and center. Since Malay patients were just as likely to start recommended treatment as Chinese patients, it is unlikely that the survival difference was due to treatment compliance. Moreover, according to the work of Hill *et al*. in breast cancer patients in the United States, the disparity in the survival of different ethnicities among the older patients in Asia was not due to deviation from guideline recommended treatment^12^. Further studies to identify other factors that delay or prevent the timely receipt of recommended treatment would allow efforts to be directed towards their mitigation.

### Treatment compliance and overall survival

The high mortality rate associated with not proceeding with recommended surgery is in agreement with results reported in existing literature^10,13^. Notably, patients who did not follow recommended chemotherapy, radiotherapy or endocrine therapy were ~2 to 3 times more likely to die from breast cancer than patients who do^12–15^.

Our results are in agreement with previous studies which found increased risk of death in patients who did not start endocrine therapy^12,15^. Interestingly, while mortality rates of breast cancer patients who followed recommended endocrine therapy were lower than that of patients who did not, the curves converged and crossed at ~2.5 and ~6 years after diagnosis (i.e. mortality rate for treatment compliant patients increased and higher than non-compliant patients). Endocrine therapy is typically recommended for at least five years. While we were not able to study drug adherence in our patients, unsatisfactory adherence to treatment for the entire recommended duration is known to be associated with worse survival, which could potentially explain the observed increasing mortality rate among treatment-compliant patients in our study^28^. As such, the survival benefit of endocrine therapy compliance would better reach its full potential if common factors contributing to non-adherence, such as patient forgetfulness and concerns about adverse effects, were followed up and addressed^29^.

Chemotherapy may appear to be less effective among patients with less aggressive breast cancers, where extensive treatment was unnecessarily administered for early stage disease^30–32^. For example, in patients who have favorable tumor characteristics (ER-positive/PR-positive, HER2-negative, and without nodal involvement) who met established guidelines for the recommendation of chemotherapy, those with favorable gene-expression profile may benefit from endocrine therapy alone^33^. Herr *et al*., found that chemotherapy did not improve survival in patients with luminal A subtype, who had tumor size >20 mm and >3 positive nodes^34^. We observed that the survival benefit of starting recommended chemotherapy was more pronounced in patients with luminal A and basal proxy subtypes and starting recommended radiotherapy has the largest survival benefit in luminal B [HER2-positive] patients. We were not able to study comorbidities in our population which may have contributed to the contradictory findings with Herr *et al*. in luminal A subtype patients, as patients who are more frail are more likely to not start chemotherapy and also have worse survival.

### Age at diagnosis and recommended chemotherapy

The NCCN guidelines restrict their recommendations on chemotherapy to breast cancer patients aged <70 years^20^. In addition, clinical trials on the use of chemotherapy typically do not recruit patients older than 70 years, providing little empirical support for the use of such treatment in older patients^35^. Clinicians are less likely to recommend aggressive or invasive treatment such as chemotherapy or surgery for older women due to the increased likelihood of existing comorbidities^23,24^. Nonetheless, older patients can benefit from optimal anti-cancer therapy. For example, a previous study in stage I and II breast cancer patients found that having surgery reduced the risk of breast cancer specific death in patients aged ≥80 years^36^.

While chemotherapy compliance is generally associated with better survival, this was not the case for older patients who were noncompliant with radiotherapy. The reason for not starting recommended treatment is not known. Clinicians may at their discretion not advise guideline-recommended treatment for patients who were already in ill health. Patient level information such as comorbidities and performance status was not available as a proxy of health status at diagnosis. Hence, it is unclear whether the worse survival observed in patients who did not receive treatment was due to the lack of treatment or other causes (e.g. patients’ preference, fear of adverse effects, or the lack of support)^26,37^.

Older patients may also be more prone to side effects from treatment, such as chemotherapy-induced neutropenia, fever and infection^38^. In older patients who complied with radiotherapy, chemotherapy conferred a substantial survival advantage. While the better survival linked to treatment compliance in the older breast cancer population could be an artifact due to more favorable health status, a similar survival benefit was still seen in a subset of patients who survived at least two years after diagnosis. This suggests that with close monitoring of patient response and the use of prophylactic agents such as granulocyte colony-stimulating factor to prevent adverse side effects, older patients too, may benefit from appropriate chemotherapy treatment^39^. Nonetheless, this result is noteworthy because women over 70 years of age comprise 13% of all breast cancer patients. Mortality rate is also highest for this age group.

Our study is not without limitations. Our classification of treatment was largely based on international guidelines which changes over time. However, most treatment modality and indication has seen small changes within our study period 2005–2015^40^. Our results on overall survival may not be generalizable to the women diagnosed between 2011 and 2015 due to the shorter follow-up time. However, stratified analysis by year of diagnosis (2005–2010 and 2011–2015, Supplementary Table 3) resulted in estimates of the adjusted hazard ratios that were similar with those of 2005–2015 in Table 4. The introduction of targeted therapy, which is commonly administered to HER2 positive patients on chemotherapy, during this period (2005–2015) could not be accounted for. This may lead to an overestimation of the benefit of chemotherapy in our study. In an attempt to mitigate this influence, we performed stratified analysis by proxy subtype. We were not able to account for potential survival differences due to disparity in treatment intensity and toxicity. However, our result is likely to be biased towards the null, as the premature stopping of treatment, dose reduction and adverse drug reactions will reduce survival in the group of patients who started treatment. In addition, the evaluation of endocrine therapy impact on survival can be improved by studying the 5 year adherence of treatment instead of only the start of treatment. Information on comorbidities was not available, which may have result in contraindication of treatments and worse survival in patients who did not start treatment. Here, we partially accounted for comorbidities in patients aged >70 who would be more affected by comorbidities. There was no appreciable difference in the results (apart from uptake in surgery), when we excluded those who survived >2 years post-diagnosis in patients aged >70. The socioeconomic status and health seeking behavior of patients attending restructured hospitals (patients are eligible for subsidized care) may be different from patients attending private hospitals. However, it is recognized that the majority (~80%) of Singaporean’s attend restructured hospitals and healthcare policies are targeted at these patients. Being geographically small with an extensive public transport system, refusal of recommended treatment due to inaccessibility is less common than in areas where specialized centers may be far from residential districts^41^.

## Conclusion

Noncompliance with recommended treatment was associated with worse survival (HR between 1.5 and 6.4). A survival benefit was observed when older breast cancer patients were treated with chemotherapy otherwise recommended only for younger patients (<70 years), suggesting that older patients may benefit from similar recommendations. Proxy subtype, pre-surgical tumor size, nodal stage, age at diagnosis and year of diagnosis were associated with treatment compliance. Our results highlight the importance of following appropriate treatment as recommended by current guidelines.

## Supplementary information

Supplementary document.

## Data Availability

Data used in this study is de-identified. Data is available upon request from Ms Yen Shing Yeoh (email: yen_shing_yeoh@nuhs.edu.sg).
