# Supplementary material for: Impact of deviation from guideline recommended treatment on breast cancer survival in Asia
Source: Sci Rep. 2020 Jan 28;10:1330. doi: 10.1038/s41598-020-58007-5 (PMC6987174; doi:10.1038/s41598-020-58007-5)
Supplement: Supplementary file 1 — Supplementary document. [file 41598_2020_58007_MOESM1_ESM.docx]

**Impact of deviation from guideline recommended treatment on breast cancer survival in Asia**

Short title: Impact of deviation from recommended treatment on survival

Peh Joo Ho^1,2^ ho_peh_joo@gis.a-star.edu.sg

Samuel Guan Wei Ow^3^ samuel_ow@nuhs.edu.sg

Yirong Sim^4,5^ sim.yirong@singhealth.com.sg

Jenny Liu^2^ ephliuj@nus.edu.sg

Swee Ho Lim^6^ lim.swee.ho@singhealth.com.sg

Ern Yu Tan^7^ ern_yu_tan@ttsh.com.sg

Su-Ming Tan^8^ tan.su.ming@singhealth.com.sg

Soo Chin Lee^3^ [csilsc@nus.edu.sg](mailto:csilsc@nus.edu.sg)

Veronique Kiak-Mien Tan^4,5^ veronique.tan.k.m@singhealth.com.sg

Yoon-Sim Yap^9^ yap.yoon.sim@singhealth.com.sg

Wen Yee Chay^9^ chay.wen.yee@singhealth.com.sg

Benita Kiat Tee Tan^4,5^ benita.tan.k.t@singhealth.com.sg

Fuh Yong Wong^4^ wong.fuh.yong@singhealth.com.sg

* Jingmei Li^1,10,11^ lijm1@gis.a-star.edu.sg

* Mikael Hartman^2,11^ ephbamh@nus.edu.sg

* These authors share senior authorship.

^1^ Human Genetics, Genome Institute of Singapore, Singapore 138672, Singapore

^2^ Saw Swee Hock School of Public Health, National University of Singapore, National University Health System, Singapore 117549, Singapore

^3^ Department of Haematology – Oncology, National University Cancer Institute Singapore (NCIS), Singapore 119074, Singapore

^4^ Division of Surgical Oncology, National Cancer Centre Singapore, Singapore, Singapore

^5^ Department of General Surgery, Singapore General Hospital, Singapore, Singapore

^6^ KK Women’s and Children’s Hospital, KK Breast Department, Singapore 229899, Singapore

^7^ Department of General Surgery, Tan Tock Seng Hospital, Singapore 308433, Singapore

^8^ Division of Breast Surgery, Department of Surgery, Changi General Hospital, Singapore, Singapore

^9^ Division of Medical Oncology, National Cancer Centre Singapore, Singapore, Singapore

^10^ Karolinska Institutet, Department of Medical Epidemiology and Biostatistics, Box 281, 171 77 Stockholm, Sweden

^11^ Department of Surgery, University Surgical Cluster, National University Hospital, Singapore, Singapore

**Supplementary Table 1:** Degrees of freedom used in flexible parametric survival models.

| **Model** | **Degrees of freedom used in baseline distribution function** | **Time varying covariate(s), and the corresponding degrees of freedom** |
| --- | --- | --- |
| **Table 4** |  |  |
| **All patients aged <70 years, unadjusted** |  |  |
| Surgery | 3 (i.e. 2 internal knots) | Surgery, 2 |
| Chemotherapy | 3 | Chemotherapy, 2 |
| Radiotherapy | 3 | Radiotherapy, 2 |
| Endocrine therapy | 3 | Endocrine therapy, 3 |
| Pre-surgical tumor size | 3 | Pre-surgical tumor size, 2 |
| TNM nodal stage | 3 | TNM nodal stage, 2 |
| Ethnicity | 3 | - |
| Age at diagnosis, years | 3 | - |
| Year of diagnosis | 3 | - |
| Center | 3 | - |
|  |  |  |
| **All patients aged <70 years, adjusted** | 3 | Surgery, 1  Radiotherapy, 1  Endocrine therapy, 1  Pre-surgical tumor size, 1 |
|  |  |  |
| **Luminal A, unadjusted** |  |  |
| Surgery | 1 | Surgery, 1 |
| Chemotherapy | 1 | Chemotherapy, 1 |
| Radiotherapy | 1 | Radiotherapy, 1 |
| Endocrine therapy | 1 | Endocrine therapy, 3 |
| Pre-surgical tumor size | 1 | - |
| TNM nodal stage | 1 | TNM nodal stage, 1 |
| Ethnicity | 1 | - |
| Age at diagnosis, years | 1 | Age at diagnosis, 2 |
| Year of diagnosis | 1 | - |
| Center | 1 | - |
|  |  |  |
| **Luminal A, adjusted** | 1 | Surgery,1  Chemotherapy,1  Radiotherapy, 1 Endocrine therapy, 3  TNM nodal stage, 1  Age at diagnosis, 2 |
|  |  |  |
| **Luminal B (HER2-negative), unadjusted** |  |  |
| Surgery | 1 | Surgery, 1 |
| Chemotherapy | 1 | - |
| Radiotherapy | 1 | - |
| Endocrine therapy | 1 | Endocrine therapy, 1 |
| Pre-surgical tumor size | 1 | Pre-surgical tumor size, 3 |
| TNM nodal stage | 1 | TNM nodal stage, 2 |
| Ethnicity | 1 | - |
| Age at diagnosis, years | 1 | - |
| Year of diagnosis | 1 | - |
| Center | 1 | - |
|  |  |  |
| **Luminal B (HER2-negative), adjusted** | 1 | Surgery,1  Endocrine therapy, 1 Pre-surgical tumor size, 2  TNM nodal stage, 2 |
|  |  |  |
| **Luminal B (HER2-positive), unadjusted** |  |  |
| Surgery | 1 | Surgery, 1 |
| Chemotherapy | 1 | Chemotherapy, 1 |
| Radiotherapy | 1 | - |
| Endocrine therapy | 1 | Endocrine therapy, 1 |
| Pre-surgical tumor size | 2 | - |
| TNM nodal stage | 1 | - |
| Ethnicity | 1 | - |
| Age at diagnosis, years | 1 | - |
| Year of diagnosis | 1 | - |
| Center | 1 | Center, 1 |
|  |  |  |
| **Luminal B (HER2-positive), adjusted** | 1 | Surgery, 1 Chemotherapy, 1 Endocrine therapy, 1 Center, 1 |
|  |  |  |
| **HER2-enriched, unadjusted** |  |  |
| Surgery | 1 | Surgery, 1 |
| Chemotherapy | 3 | Chemotherapy, 1 |
| Radiotherapy | 3 | - |
| Pre-surgical tumor size | 3 | Pre-surgical tumor size, 1 |
| TNM nodal stage | 3 | - |
| Ethnicity | 3 | - |
| Age at diagnosis, years | 3 | - |
| Year of diagnosis | 3 | - |
| Center | 3 | - |
|  |  |  |
| **HER2-enriched, adjusted** | 3 | Surgery, 1  Chemotherapy, 1  Pre-surgical tumor size, 1 |
|  |  |  |
| **Basal, unadjusted** |  |  |
| Surgery | 2 | Surgery, 2 |
| Chemotherapy | 2 | Chemotherapy, 2 |
| Radiotherapy | 2 | Radiotherapy,2 |
| Pre-surgical tumor size | 2 | Pre-surgical tumor size, 1 |
| TNM nodal stage | 2 | TNM nodal stage, 2 |
| Ethnicity | 2 | - |
| Age at diagnosis, years | 2 | - |
| Year of diagnosis | 2 | 1 |
| Center | 2 | - |
|  |  |  |
| **Basal, adjusted** | 2 | Chemotherapy, 2 Radiotherapy,2 |
|  |  |  |
| **Supplementary Table 2** |  |  |
| **All, unadjusted** |  |  |
| Chemotherapy | 1 | - |
| Surgery | 1 | Surgery, 1 |
| Radiotherapy | 1 | - |
| Endocrine therapy | 1 | Endocrine therapy, 1 |
| Pre-surgical tumor size | 1 | Pre-surgical tumor size, 1 |
| TNM nodal stage | 1 | TNM nodal stage, 1 |
| Proxy subtype | 1 | Proxy subtype, 1 |
| Ethnicity | 1 | - |
| Age at diagnosis, years | 1 | Age at diagnosis, 1 |
| Year of diagnosis | 1 | Year of diagnosis, 1 |
| Grade | 1 | Grade, 2 |
| Estrogen receptor status | 1 | Estrogen receptor status, 1 |
| Progesterone receptor status | 1 | Progesterone receptor status, 1 |
| HER2 status | 1 | - |
| Center | 1 | - |
|  |  |  |
| **All, adjusted for proxy subtype** | 1 | Surgery, 1  Endocrine therapy, 1  Pre-surgical tumor size, 1  TNM nodal stage, 1  Proxy subtype, 1  Age at diagnosis, 1  Year of diagnosis, 1 |
|  |  |  |
| **All, adjusted for grade, estrogen receptor, progesterone receptor and HER2 status** | 1 | Surgery, 1  Endocrine therapy, 1  Pre-surgical tumor size, 1  TNM nodal stage, 1  Age at diagnosis, 1  Grade, 2  Estrogen receptor status, 1  Progesterone receptor status, 1 |
|  |  |  |
| **Survived at least two years, unadjusted** |  |  |
| Chemotherapy | 3 | - |
| Surgery | 3 | - |
| Radiotherapy | 3 | - |
| Endocrine therapy | 3 | - |
| Pre-surgical tumor size | 3 | - |
| TNM nodal stage | 3 | - |
| Proxy subtype | 3 | Proxy subtype, 1 |
| Ethnicity | 3 | - |
| Age at diagnosis, years | 3 | - |
| Year of diagnosis | 3 | - |
| Grade | 3 | Grade, 1 |
| Estrogen receptor status | 3 | - |
| Progesterone receptor status | 3 | - |
| HER2 status | 3 | - |
|  |  |  |
| **Survived at least two years, adjusted for proxy subtype** | 3 | Proxy subtype, 1 |
|  |  |  |
| **Survived at least two years, adjusted for grade, estrogen receptor, progesterone receptor and HER2 status** | 3 | - |
|  |  |  |
| **Figure 1** |  |  |
| (a) Surgery | 3 | Surgery, 3 |
| (b) Chemotherapy | 3 | Chemotherapy, 3 |
| (c) Radiotherapy | 3 | Radiotherapy, 2 |
| (d) Endocrine therapy | 3 | Endocrine therapy, 3 |

**Supplementary Table 2:** The associations between demographic, clinical, and treatment variables and overall survival, in women aged 70 years. HR: Hazards ratio, CI: Confidence interval. ^Additionally adjusted for site.

|  | **All patients** | | | **Survived at least two years** | | |
| --- | --- | --- | --- | --- | --- | --- |
|  | **Unadjusted** | **Adjusted^** | **Adjusted^** | **Unadjusted** | **Adjusted^** | **Adjusted^** |
|  | **HR (95%CI)** | **HR (95%CI)** | **HR (95%CI)** | **HR (95%CI)** | **HR (95%CI)** | **HR (95%CI)** |
| **Surgery** |  |  |  |  |  |  |
| Yes | 1.00 (Reference) | 1.00 (Reference) | 1.00 (Reference) | 1.00 (Reference) | 1.00 (Reference) | 1.00 (Reference) |
| No/ not recommended | 4.11 (3.53 – 4.79) | 1.74 (1.33 – 2.29) | 1.82 (1.36 – 2.42) | 2.38 (1.97 – 2.89) | 1.30 (0.92 – 1.84) | 1.25 (0.87 – 1.81) |
|  |  |  |  |  |  |  |
| **Chemotherapy** |  |  |  |  |  |  |
| Yes | 1.00 (Reference) | 1.00 (Reference) | 1.00 (Reference) | 1.00 (Reference) | 1.00 (Reference) | 1.00 (Reference) |
| No | 1.38 (1.11 – 1.71) | 1.31 (1.03 – 1.66) | 1.32 (1.04 – 1.68) | 1.46 (1.11 – 1.93) | 1.43 (1.05 – 1.95) | 1.43 (1.05 – 1.95) |
| Not recommended | 0.87 (0.69 – 1.08) | 1.21 (0.87 – 1.68) | 1.38 (1.01 – 1.89) | 0.87 (0.66 – 1.16) | 1.29 (0.85 – 1.95) | 1.40 (0.94 – 2.09) |
|  |  |  |  |  |  |  |
| **Radiotherapy** |  |  |  |  |  |  |
| Yes | 1.00 (Reference) | 1.00 (Reference) | 1.00 (Reference) | 1.00 (Reference) | 1.00 (Reference) | 1.00 (Reference) |
| No | 1.70 (1.38 – 2.10) | 1.64 (1.30 – 2.07) | 1.66 (1.31 – 2.09) | 1.56 (1.21 – 2.01) | 1.64 (1.23 – 2.18) | 1.70 (1.28 – 2.26) |
| Not recommended | 0.74 (0.61 – 0.89) | 0.84 (0.66 – 1.08) | 0.84 (0.65 – 1.08) | 0.82 (0.65 – 1.02) | 1.02 (0.76 – 1.38) | 0.99 (0.73 – 1.34) |
|  |  |  |  |  |  |  |
| **Endocrine therapy** |  |  |  |  |  |  |
| Yes | 1.00 (Reference) | 1.00 (Reference) | 1.00 (Reference) | 1.00 (Reference) | 1.00 (Reference) | 1.00 (Reference) |
| No | 1.49 (1.14 – 1.95) | 1.57 (1.20 – 2.07) | 1.53 (1.16 – 2.01) | 0.75 (0.51 – 1.10) | 0.84 (0.60 – 1.17) | 0.89 (0.64 – 1.24) |
| Not recommended | 2.59 (2.18 – 3.08) | 2.54 (1.68 – 3.83) | 0.72 (0.46 – 1.13) | 1.70 (1.37 – 2.12) | 1.37 (0.79 – 2.37) | 0.57 (0.33 – 0.99) |
|  |  |  |  |  |  |  |
| **Pre-surgical tumor size** |  |  |  |  |  |  |
| ≤20mm | 1.00 (Reference) | 1.00 (Reference) | 1.00 (Reference) | 1.00 (Reference) | 1.00 (Reference) | 1.00 (Reference) |
| 21 – 50mm | 1.95 (1.59 – 2.39) | 1.49 (1.21 – 1.84) | 1.47 (1.19 – 1.81) | 1.92 (1.57 – 2.36) | 1.58 (1.28 – 1.96) | 1.55 (1.25 – 1.91) |
| >50mm | 2.01 (1.53 – 2.63) | 1.44 (1.09 – 1.90) | 1.36 (1.03 – 1.80) | 2.04 (1.53 – 2.72) | 1.64 (1.21 – 2.24) | 1.56 (1.15 – 2.13) |
| Attached to chest wall | 3.55 (2.74 – 4.61) | 1.63 (1.23 – 2.16) | 1.63 (1.23 – 2.16) | 2.83 (2.12 – 3.79) | 1.83 (1.32 – 2.53) | 1.78 (1.28 – 2.46) |
|  |  |  |  |  |  |  |
| **TNM nodal stage** |  |  |  |  |  |  |
| N0 | 1.00 (Reference) | 1.00 (Reference) | 1.00 (Reference) | 1.00 (Reference) | 1.00 (Reference) | 1.00 (Reference) |
| N1 | 0.79 (0.63 – 0.99) | 0.77 (0.57 – 1.03) | 0.79 (0.59 – 1.05) | 1.07 (0.86 – 1.34) | 0.82 (0.60 – 1.13) | 0.80 (0.58 – 1.10) |
| N2 | 0.88 (0.63 – 1.22) | 0.92 (0.66 – 1.29) | 0.96 (0.69 – 1.34) | 1.51 (1.15 – 1.98) | 1.28 (0.89 – 1.84) | 1.29 (0.90 – 1.85) |
| N3 | 1.89 (1.49 – 2.40) | 1.87 (1.40 – 2.50) | 1.87 (1.40 – 2.50) | 2.31 (1.76 – 3.03) | 1.96 (1.36 – 2.81) | 1.90 (1.33 – 2.73) |
|  |  |  |  |  |  |  |
| **Ethnicity** |  |  |  |  |  |  |
| Chinese | 1.00 (Reference) | 1.00 (Reference) | 1.00 (Reference) | 1.00 (Reference) | 1.00 (Reference) | 1.00 (Reference) |
| Malay | 1.79 (1.43 – 2.24) | 1.77 (1.40 – 2.24) | 1.76 (1.39 – 2.23) | 1.50 (1.10 – 2.05) | 1.49 (1.08 – 2.05) | 1.53 (1.11 – 2.11) |
| Indian | 1.21 (0.93 – 1.57) | 1.42 (1.09 – 1.85) | 1.45 (1.11 – 1.90) | 1.33 (0.97 – 1.81) | 1.60 (1.16 – 2.20) | 1.69 (1.23 – 2.33) |
| Other | 2.22 (1.65 – 3.00) | 2.05 (1.51 – 2.79) | 1.94 (1.42 – 2.65) | 2.09 (1.41 – 3.10) | 1.96 (1.31 – 2.94) | 1.91 (1.27 – 2.86) |
|  |  |  |  |  |  |  |
| **Age at diagnosis, years** | 1.09 (1.08 – 1.10) | 1.06 (1.05 – 1.07) | 1.06 (1.05 – 1.08) | 1.07 (1.06 – 1.09) | 1.06 (1.04 – 1.07) | 1.06 (1.04 – 1.08) |
|  |  |  |  |  |  |  |
| **Year of diagnosis** |  |  |  |  |  |  |
| 2005-2010 | 1.00 (Reference) | 1.00 (Reference) | 1.00 (Reference) | 1.00 (Reference) | 1.00 (Reference) | 1.00 (Reference) |
| 2011-2015 | 1.05 (0.91 – 1.22) | 0.95 (0.82 – 1.11) | 0.98 (0.84 – 1.14) | 0.79 (0.65 – 0.95) | 0.74 (0.61 – 0.90) | 0.75 (0.62 – 0.91) |
|  |  |  |  |  |  |  |
| **Proxy subtype** |  |  |  |  |  |  |
| Luminal A | 1.00 (Reference) | 1.00 (Reference) | – | 1.00 (Reference) | 1.00 (Reference) | – |
| Luminal B, HER2-negative | 1.93 (1.46 – 2.56) | 1.62 (1.19 – 2.20) |  | 2.38 (1.75 – 3.22) | 1.92 (1.36 – 2.71) |  |
| Luminal B, HER2-positive | 1.79 (1.32 – 2.43) | 1.33 (0.95 – 1.85) |  | 2.27 (1.64 – 3.12) | 1.73 (1.20 – 2.48) |  |
| HER2-enriched | 3.79 (2.90 – 4.95) | 1.38 (0.87 – 2.21) |  | 2.47 (1.71 – 3.56) | 1.63 (0.86 – 3.09) |  |
| Basal | 3.42 (2.63 – 4.43) | 1.37 (0.87 – 2.16) |  | 3.15 (2.31 – 4.30) | 2.05 (1.13 – 3.74) |  |
|  |  |  |  |  |  |  |
| **Grade** |  |  |  |  |  |  |
| Well-differentiated | 1.00 (Reference) | – | 1.00 (Reference) | 1.00 (Reference) | – | 1.00 (Reference) |
| Moderately-differentiated | 1.13 (0.85 – 1.50) |  | 0.99 (0.74 – 1.31) | 1.80 (1.24 – 2.62) |  | 1.39 (1.03 – 1.87) |
| Poorly-differentiated | 2.32 (1.78 – 3.03) |  | 1.46 (1.08 – 1.97) | 3.65 (2.54 – 5.24) |  | 1.93 (1.39 – 2.67) |
|  |  |  |  |  |  |  |
| **Estrogen receptor status** |  |  |  |  |  |  |
| Positive | 1.00 (Reference) | – | 1.00 (Reference) | 1.00 (Reference) | – | 1.00 (Reference) |
| Negative | 0.42 (0.35 – 0.49) |  | 0.37 (0.25 – 0.55) | 0.60 (0.50 – 0.73) |  | 0.50 (0.31 – 0.80) |
|  |  |  |  |  |  |  |
| **Progesterone receptor status** |  |  |  |  |  |  |
| Positive | 1.00 (Reference) | – | 1.00 (Reference) | 1.00 (Reference) | – | 1.00 (Reference) |
| Negative | 0.49 (0.42 – 0.58) |  | 0.66 (0.52 – 0.84) | 0.63 (0.53 – 0.75) |  | 0.66 (0.52 – 0.84) |
|  |  |  |  |  |  |  |
| **HER2 status** |  |  |  |  |  |  |
| Positive | 1.00 (Reference) | – | 1.00 (Reference) | 1.00 (Reference) | – | 1.00 (Reference) |
| Negative | 1.45 (1.23 – 1.72) |  | 0.99 (0.82 – 1.20) | 1.36 (1.10 – 1.68) |  | 1.00 (0.79 – 1.26) |

**Supplementary Table 3:** The associations between demographic, clinical, and treatment variables and overall survival using the flexible parametric survival model, stratified by year of diagnosis (2005-2010, 2011-2015), in patients aged <70 years. Degrees of freedom used in the flexible parametric survival models are shown in **Supplementary Table 1** (All patients aged <70 years, unadjusted; All patients aged <70 years, adjusted). HR: Hazards ratio, CI: Confidence interval. ^Adjusted for all variables listed in the table and site.

|  | **Year of diagnosis (2005-2010)** | | | | **Year of diagnosis (2011-2015)** | | | |
| --- | --- | --- | --- | --- | --- | --- | --- | --- |
|  | **Alive** | **Dead** | **Unadjusted** | **Adjusted^** | **Alive** | **Dead** | **Unadjusted** | **Adjusted^** |
|  | **n (%)** | **n (%)** | **HR (95%CI)** | **HR (95%CI)** | **n (%)** | **n (%)** | **HR (95%CI)** | **HR (95%CI)** |
| **Surgery** |  |  |  |  |  |  |  |  |
| Yes | 7282 (86) | 1213 (14) | 1.00 (Reference) | 1.00 (Reference) | 6549 (93) | 467 (7) | 1.00 (Reference) | 1.00 (Reference) |
| No | 395 (54) | 331 (46) | 4.70 (4.15 – 5.33) | 2.42 (1.76 – 3.34) | 281 (57) | 208 (43) | 13.62 (11.36 – 16.33) | 2.29 (1.65 – 3.19) |
|  |  |  |  |  |  |  |  |  |
| **Chemotherapy** |  |  |  |  |  |  |  |  |
| Yes | 3253 (79) | 848 (21) | 1.00 (Reference) | 1.00 (Reference) | 2797 (89) | 354 (11) | 1.00 (Reference) | 1.00 (Reference) |
| No | 961 (74) | 332 (26) | 1.48 (1.30 – 1.68) | 1.25 (1.08 – 1.45) | 981 (86) | 155 (14) | 1.58 (1.29 – 1.94) | 1.28 (1.03 – 1.58) |
| Not recommended | 3463 (90) | 364 (10) | 0.45 (0.39 – 0.51) | 0.66 (0.56 – 0.78) | 3052 (95) | 166 (5) | 0.51 (0.41 – 0.62) | 0.70 (0.55 – 0.89) |
|  |  |  |  |  |  |  |  |  |
| **Radiotherapy** |  |  |  |  |  |  |  |  |
| Yes | 3673 (85) | 646 (15) | 1.00 (Reference) | 1.00 (Reference) | 2935 (93) | 220 (7) | 1.00 (Reference) | 1.00 (Reference) |
| No | 497 (72) | 192 (28) | 2.47 (2.08 – 2.94) | 2.25 (1.88 – 2.70) | 515 (88) | 71 (12) | 2.12 (1.50 – 2.98) | 2.13 (1.58 – 2.88) |
| Not recommended | 2871 (91) | 291 (9) | 0.69 (0.59 – 0.81) | 1.39 (1.14 – 1.69) | 2591 (96) | 98 (4) | 0.59 (0.43 – 0.80) | 1.26 (0.90 – 1.78) |
|  |  |  |  |  |  |  |  |  |
| **Endocrine therapy** |  |  |  |  |  |  |  |  |
| Yes | 4322 (86) | 727 (14) | 1.00 (Reference) | 1.00 (Reference) | 3904 (93) | 300 (7) | 1.00 (Reference) | 1.00 (Reference) |
| No | 586 (84) | 108 (16) | 1.42 (1.15 – 1.76) | 1.46 (1.18 – 1.81) | 575 (93) | 45 (7) | 1.37 (0.98 – 1.91) | 1.41 (1.01 – 1.97) |
| Not recommended | 1410 (78) | 393 (22) | 2.03 (1.77 – 2.32) | 1.46 (1.28 – 1.66) | 1215 (87) | 187 (13) | 2.41 (1.96 – 2.96) | 1.79 (1.47 – 2.17) |
|  |  |  |  |  |  |  |  |  |
| **Pre-surgical tumor size** |  |  |  |  |  |  |  |  |
| ≤20mm | 3819 (93) | 292 (7) | 1.00 (Reference) | 1.00 (Reference) | 3035 (97) | 103 (3) | 1.00 (Reference) | 1.00 (Reference) |
| 21 – 50mm | 2264 (81) | 535 (19) | 2.91 (2.48 – 3.41) | 1.73 (1.49 – 2.01) | 2050 (91) | 197 (9) | 2.74 (2.04 – 3.68) | 1.67 (1.30 – 2.14) |
| >50mm | 734 (75) | 239 (25) | 4.16 (3.46 – 5.01) | 2.56 (2.12 – 3.07) | 780 (88) | 103 (12) | 3.53 (2.50 – 4.98) | 2.02 (1.50 – 2.71) |
| Attached to chest wall | 174 (51) | 164 (49) | 10.29 (8.40 – 12.61) | 4.30 (3.49 – 5.29) | 240 (70) | 105 (30) | 13.11 (9.59 – 17.94) | 3.51 (2.60 – 4.74) |
|  |  |  |  |  |  |  |  |  |
| **TNM nodal stage** |  |  |  |  |  |  |  |  |
| N0 | 5130 (92) | 458 (8) | 1.00 (Reference) | 1.00 (Reference) | 4493 (97) | 152 (3) | 1.00 (Reference) | 1.00 (Reference) |
| N1 | 1359 (81) | 309 (19) | 2.20 (1.86 – 2.60) | 1.61 (1.30 – 1.99) | 1197 (91) | 120 (9) | 2.73 (2.01 – 3.72) | 1.88 (1.32 – 2.68) |
| N2 | 536 (70) | 234 (30) | 3.99 (3.35 – 4.75) | 2.92 (2.35 – 3.63) | 442 (86) | 71 (14) | 3.11 (1.96 – 4.93) | 2.89 (1.97 – 4.22) |
| N3 | 263 (56) | 210 (44) | 7.02 (5.89 – 8.35) | 4.28 (3.42 – 5.34) | 295 (74) | 106 (26) | 8.73 (6.42 – 11.88) | 5.11 (3.56 – 7.34) |
|  |  |  |  |  |  |  |  |  |
| **Ethnicity** |  |  |  |  |  |  |  |  |
| Chinese | 6310 (85) | 1109 (15) | 1.00 (Reference) | 1.00 (Reference) | 5410 (92) | 460 (8) | 1.00 (Reference) | 1.00 (Reference) |
| Malay | 749 (73) | 282 (27) | 1.99 (1.75 – 2.27) | 1.55 (1.35 – 1.77) | 761 (83) | 154 (17) | 2.27 (1.89 – 2.73) | 1.65 (1.37 – 1.98) |
| Indian | 390 (78) | 112 (22) | 1.58 (1.30 – 1.92) | 1.18 (0.97 – 1.44) | 415 (91) | 43 (9) | 1.23 (0.90 – 1.69) | 1.06 (0.77 – 1.45) |
| Other | 228 (85) | 41 (15) | 1.10 (0.81 – 1.51) | 1.10 (0.81 – 1.51) | 244 (93) | 18 (7) | 0.99 (0.62 – 1.59) | 1.08 (0.67 – 1.74) |
|  |  |  |  |  |  |  |  |  |
| **Age at diagnosis, years** |  |  |  |  |  |  |  |  |
| <50 | 3516 (86) | 564 (14) | 0.72 (0.65 – 0.80) | 0.76 (0.68 – 0.84) | 2425 (93) | 194 (7) | 0.78 (0.66 – 0.92) | 0.86 (0.72 – 1.01) |
| 50-69 | 4161 (81) | 980 (19) | 1.00 (Reference) | 1.00 (Reference) | 4405 (90) | 481 (10) | 1.00 (Reference) | 1.00 (Reference) |

**Supplementary Figure 1: Flow diagrams of derivation of analytical cohorts.**

**Breast cancer patients diagnosed between 2005 and 2015
(*n*=20,999)**

**Excluded (*n*=1,758)**

- Males (*n*=26)
- Stage IV (*n*=1,644)
- Missing information on death (*n*=82)
- Missing age (*n*=6)

**Female non-metastatic breast cancer patients with information on mortality
(*n*=19,241)**
